# Interleukin-1β induces and accelerates human endometrial stromal cell senescence and impairs decidualization via the c-Jun N-terminal kinase pathway

**DOI:** 10.1038/s41420-024-02048-6

**Published:** 2024-06-15

**Authors:** Robert N. Taylor, Sarah L. Berga, Eric Zou, Jacara Washington, Sunyangzi Song, Brandon J. Marzullo, Indrani C. Bagchi, Milan K. Bagchi, Jie Yu

**Affiliations:** 1 Department of Obstetrics and Gynecology, Jacobs School of Medicine and Biomedical Sciences, University at Buffalo, Buffalo, NY USA; 2 Department of Pathology and Anatomical Sciences, Jacobs School of Medicine and Biomedical Sciences, University at Buffalo, Buffalo, NY USA; 3 Department of Obstetrics and Gynecology, Wake Forest School of Medicine, Winston-Salem, NC USA; 4 Genomics and Bioinformatics Core, Jacobs School of Medicine and Biomedical Sciences, University at Buffalo, Buffalo, NY USA; 5 Departments of Comparative Biosciences, University of Illinois, Urbana/Champaign, Urbana, IL USA; 6 Molecular and Integrative Physiology, University of Illinois, Urbana/Champaign, Urbana, IL USA

**Keywords:** Cell death, Ageing

## Abstract

As the mean age of first-time mothers increases in the industrialized world, inquiries into causes of human reproductive senescence have followed. Rates of ovulatory dysfunction and oocyte aneuploidy parallel chronological age, but poor reproductive outcomes in women older than 35 years are also attributed to endometrial senescence. The current studies, using primary human endometrial stromal cell (ESC) cultures as an in vitro model for endometrial aging, characterize the proinflammatory cytokine, IL-1β-mediated and passage number-dependent effects on ESC phenotype. ESC senescence was accelerated by incubation with IL-1β, which was monitored by RNA sequencing, ELISA, immunocytochemistry and Western blotting. Senescence associated secreted phenotype (SASP) proteins, IL-1β, IL-6, IL-8, TNF-α, MMP3, CCL2, CCL5, and other senescence-associated biomarkers of DNA damage (p16, p21, HMGB1, phospho-γ-histone 2 A.X) were noted to increase directly in response to 0.1 nM IL-1β stimulation. Production of the corresponding SASP proteins increased further following extended cell passage. Using enzyme inhibitors and siRNA interference, these effects of IL-1β were found to be mediated via the c-Jun N-terminal kinase (JNK) signaling pathway. Hormone-induced ESC decidualization, classical morphological and biochemical endocrine responses to estradiol, progesterone and cAMP stimulation (prolactin, IGFBP-1, IL-11 and VEGF), were attenuated *pari passu* with prolonged ESC passaging. The kinetics of differentiation responses varied in a biomarker-specific manner, with IGFBP-1 and VEGF secretion showing the largest and smallest reductions, with respect to cell passage number. ESC hormone responsiveness was most robust when limited to the first six cell passages. Hence, investigation of ESC cultures as a decidualization model should respect this limitation of cell aging. The results support the hypotheses that “inflammaging” contributes to endometrial senescence, disruption of decidualization and impairment of fecundity. IL-1β and the JNK signaling pathway are pathogenetic targets amenable to pharmacological correction or mitigation with the potential to reduce endometrial stromal senescence and enhance uterine receptivity.

## Introduction

Advanced maternal age and reproductive senescence reduce pregnancy success and quality of life in affected women, their families and society. Postponing reproduction raises the likelihood of unwanted childlessness and, even when successful, poses a higher risk for clinical pregnancy complications, including fetal aneuploidy and preeclampsia [1]. While most research on reproductive aging in women has focused on epigenetic oocyte damage [2], endometrial senescence remains largely understudied [3, 4]. Assessment of serum glycodelin-A (PP14), an endometrial glycoprotein [5], failed to detect substantive changes in this biomarker among women 20–30 years *vs*. those 40–50 years old [6]. However, comprehensive microarray analyses of endometrial stromal cell (ESC) mRNAs derived from perimenopausal women exhibited a significantly altered transcriptome compared to ESC from premenopausal women [7].

In hemochorial placental species (*viz*, women, non-human old-world primates, bats, and mice), blastocyst nidation occurs within a narrow window of uterine receptivity synchronized to embryo development. In women, the “open” endometrial interval is regulated by ovarian steroid hormones, locally expressed growth factors and cytokines [8]. The implantation window is vulnerable to perturbation. A critical manifestation of receptive human endometrium is the decidual response, differentiation of endometrial mesenchyme into a secretory stroma supportive of placentation [9]. Biomarker proteins associated with receptive endometrium include prolactin [10]; IGFBP1 [11], IL-11 [12] and VEGF [13], among others. Another phenotypic change associated with decidualized ESCs is mesenchymal-to-epithelial transformation (MET), a cytological program shared with angiogenesis and tumorigenesis [14, 15].

Replicative senescence is an irreversible response to excessive cellular stress that limits cell longevity. It is characterized by resistance to mitogenesis and permanent cell cycle arrest. Established biomarkers of senescence associated secreted phenotype (SASP: IL-1β, IL-6, IL-8, TNF-α, MMP3, CCL2, CCL5) and nuclear protein indicators of DNA damage (p16, p21, HMGB1, phospho-γ-histone 2 A.X). Impaired decidualization in ESC derived from women with endometriosis [16, 17] and polycystic ovary disease (PCOS) [18, 19] have been reported. IL-1β can induce intrauterine inflammation and impair ESC decidualization and function [20–23]. Indeed, inverse correlations between in vitro fertilization (IVF) implantation rates and free serum [24] or intrauterine fluid IL-1β concentrations [25] are observed. In the current study we investigated whether IL-1β-treatment of primary human ESC cultures could recapitulate ESC senescence and whether its signature, so-called “inflammaging” biomarkers [26], were associated with decidual dysfunction.

## Results

### Critical role of inflammation as a modifier of ESC senescence

**Fig. 1:**
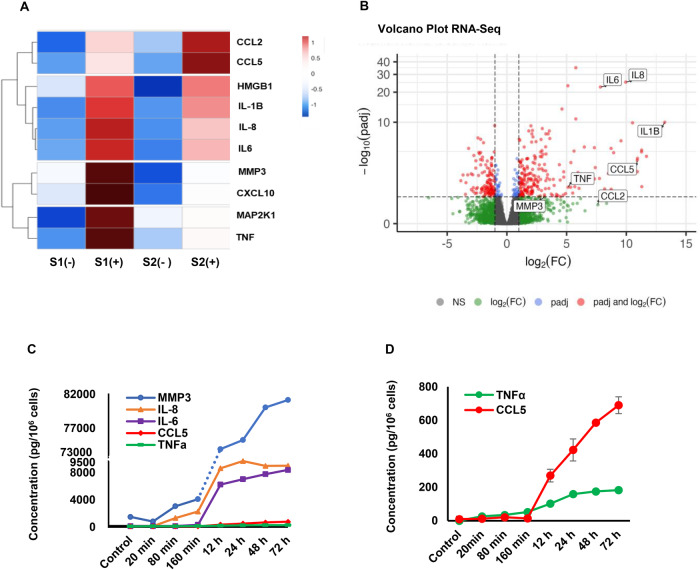
RNA sequencing (RNAseq) and ELISA assays were used to confirm the senescence induced by IL-1β. **A** The heatmap of RNA-Seq. P3 ESC from two subjects (S1, S2) were cultured without (-) or with (+) 0.1 nM IL-1β for 24 h and subjected to RNAseq for transcript profiling. The top 10 mRNAs upregulated by IL-1β are illustrated in the heat map. Red color indicates upregulated and blue color downregulated genes (fold changes shown in inset). **B** Volcano Plot RNA-Seq. A volcano plot demonstrates strong upregulation of SASP mRNA transcripts in P3 ESC from subjects S1 and S2 24 h after IL-1β treatment. IL-1β itself, IL-6, IL-8, CCL2, CCL5 and TNF-α mRNA were all increased over control and to a lesser extent, MMP3. **C** ELISA assay. ELISA of five SASP biomarker proteins in P3 ESC treated supernatants follow a time course after stimulation with IL-1β for up to 72 h. At the 72-h mark, ANOVA analysis revealed a significant overall difference (*p* < 0.001). The Scheffé Test results indicated significant differences between MMP3 and IL-8, IL-6, RANTES, and TNFα (*p* < 0.001 for all); between IL-8 and IL-6, RANTES, and TNFα (*p* > 0.05, *p* < 0.001, *p* < 0.001, respectively); and between IL-6 and RANTES, and TNFα (*p* < 0.001 for both), while for RANTES versus TNFα, there was no significant differences (*p* > 0.05). Each experiment was repeated three times. Error bars indicate SD of triplicate determinations. **D** ELISA assay. Data graphed with an expanded ordinate represent CCL5 and TNF-α, to adjust for relatively low absolute cytokine concentrations. Error bars indicate SD of triplicate determinations for CCL5. Other ELISA assays were done with duplicate samples.

IL-1β effects on gene expression were agnostically evaluated in ESC derived from different subjects (*n* = 2, S1 and S2), treated without or with 0.1 nM IL-1β for 24 h. RNAseq identified several IL-1β-induced mRNA transcripts; the 10 that were most strongly upregulated are shown in the heat map (Fig. 1A): IL-1β itself, IL-6, IL-8, HMGB1, CCL2, MMP3, CXCL10, MAP2K1, CCL5 and TNF-α. The heatmap on the left (Fig. 1A) was generated using normalized gene expression values for each gene/sample. These values were standardized to center around zero. Genes (rows) were clustered based on their Euclidean distance using the complete linkage agglomeration method. The figure was created in R using the heatmap package. As shown in the volcano plot (Fig. 1B), IL-1β treatment upregulated six SASP mRNA transcripts (IL-1β, IL-6, IL-8, CCL2, CCL5 and TNF-α) and, to a lesser extent, MMP3. The volcano plot in Fig. 1B utilized adjusted p-values and fold-changes calculated by DESeq2. The adjusted p-value cut-off was set at 0.05, and the log2 (fold-change) cut-off was set at −1 and +1. The figure was generated in R using the Enhanced Volcano package.

Senescence transcripts identified by RNAseq were confirmed at the protein level by ELISA in ESC conditioned media. MMP3, IL-6, IL-8, CCL5 and TNF-α were all stimulated in response to IL-1β (Fig. 1C). The analysis, conducted 72 hours post-treatment, compared IL-1β-treated samples with untreated controls using ANOVA, revealing a significant effect (*p* < 0.001). Subsequent post-hoc Scheffé tests identified significant differences between MMP3 and other biomarkers like IL-8, IL-6, RANTES, and TNFα (*p* < 0.001). However, no significant difference was noted between IL-8 and IL-6 (*p* > 0.05), while both CCL5 and TNFα showed significant disparities (*p* < 0.001). Comparatively, the differences for IL-6 versus RANTES and TNFα were significant (*p* < 0.001), but not between RANTES and TNFα (*p* > 0.05). These findings highlight the distinct regulation of SASP biomarkers in response to IL-1β stimulation in P3 ESCs.

**Fig. 2:**
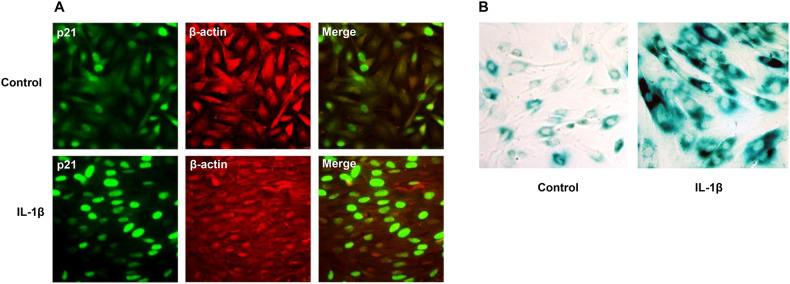
Immunofluorescence cytochemistry (IFC) and Senescence-associated-β-galactosidase(SAβG) staining were used to localize p21, β-actin and β-glacotosidase in ESC before (Control) and after IL-1β stimulation. **A** P3 ESC show increased nuclear p21 (green signal, left panel) after IL-1β-treatment. β-Actin (red) staining is more diffuse in IL-1β-treated ESC and the cellular orientation is more parallel. Merged channel images (yellow signal, right panels) are included. Magnification × 200. **B** SA-β-gal staining was used to detect β-galactosidase-positive signals. There was an intense blue color in the cytoplasm of ESC following 24 h of IL-1β treatment (right panel) compared to the untreated control (left panel). Magnification × 200.

Data in Fig. 1D are graphed with an expanded ordinate scale to resolve the lower absolute concentrations of CCL5 and TNF-α in ESC supernatants ( 800 pg/ml). Appreciable increases in biomarker protein secretion were not observed until after 80 min exposure to IL-1β. IFC experiments revealed increased p21 (green staining, Fig. 2A) accumulation in cells exposed to recombinant IL-1β for 24 h relative to controls. IFC with β-actin antibody (red staining, Fig. 2A, middle panels) showed that IL-1β induced more diffuse signal, consistent with its effect on ESC fibroblastic morphology [17]. Merged channel images show antibody overlap (Fig. 2A, right panels). β-Galactosidase staining at pH 6.0 was conducted on untreated ESC (left) and senescent ESC treated with IL-1β (right) for 24 h (Fig. 2B). The results revealed notably intense dark blue signals (Fig. 2B, right panels) indicative of cellular senescence following IL-1β treatment in ESC.

**Fig. 3:**
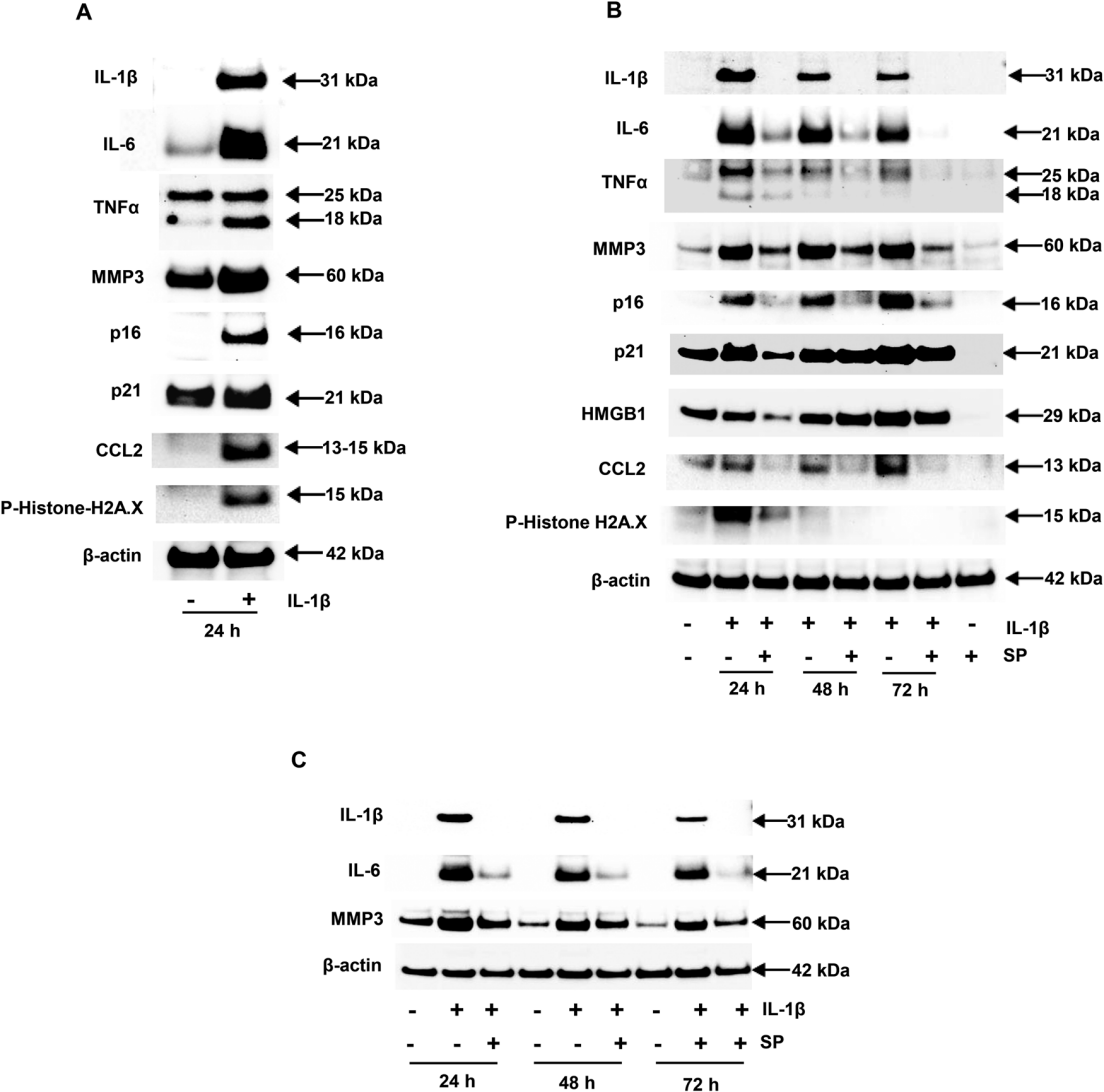
Upregulation of SASP protein biomarkers in response to IL-1β by western blot analysis. **A** After 48 h incubation, Western blotting shows that IL-1β increased IL-1β, IL-6, TNF-α, MMP3, p16, p21, CCL2 and phospho-histone H2A.X in P3 ESC. **B** Time-course experiments reveal that IL-1β upregulated IL-1β, IL-6, TNF-α, MMP3, p16, p21, HMGB1, CCL2, CCL5 and phospho-histone H2A.X at 24, 48 and 72 h (lanes 2, 4 and 6). Co-incubation in the presence of the JNK inhibitor SP blocked IL-1β-mediated upregulation at all time points (lanes 3, 5 and 7). Incubation with SP alone (lane 8) suppressed many biomarkers below basal (Control, lane 1) levels. β-Actin levels were not affected. Molecular masses (kDa) are indicated by arrows at right. **C** Based on the time course experiments, the three key senescence-associated biomarkers indicated that the baseline levels of IL-1β and IL-6 were notably low and undetectable by the western blot method at all three time points. Meanwhile, MMP3 expression levels in the untreated control group remained consistently similar across the three observed time points. β-Actin levels were not affected. Molecular masses (kDa) are indicated by arrows at right.

Western blotting of cell lysates confirmed that 24 h exposure to IL-1β upregulated senescence proteins IL-1β, IL-6, TNF-α, MMP3, p16, p21, HMGB1, CCL2, CCL5 along with phospho-histone H2A.X. β-Actin levels were not changed (Fig. 3A). Co-incubation with the JNK inhibitor SP suppressed upregulated SASP markers to near baseline levels over a 24-72 h time course (Fig. 3B, C). Notably, SP treatment alone (lane 8, Fig. 3B) reduced the levels of p21, HMGB1, CCL2 and phospho-histone H2A.X, SP alone (lane 8) reduced levels below those of untreated controls (lane 1, Fig. 3B). The latter phenomenon is in keeping with endogenous IL-1β production by ESC [17]. Additionally, analysis of three key senescence-associated biomarkers revealed that the baseline levels of IL-1β and IL-6 were notably low and remained undetectable by Western blot at all three time points examined, even under reasonable exposure times. Similarly, in the untreated control groups, MMP3 expression levels showed no significant changes across these time points (Fig. 3C).

**Fig. 4:**
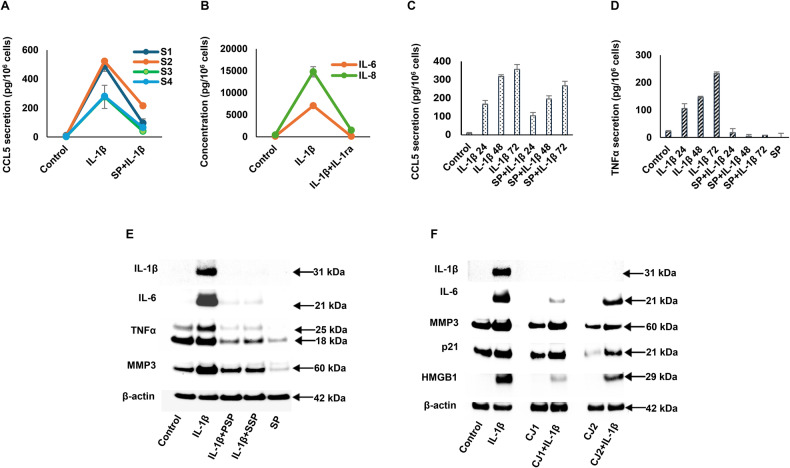
The inhibitory effects of the JNK inhibitor SP in response to IL-1β upregulation, as measured by ELISA assay and western blot analysis. **A** ELISA confirmed that CCL5 secretion by P3 ESC from each of four subjects was upregulated after 72 h of IL-1β treatment. This effect was reversed by co-incubation with SP (*p* < 0.001, ANOVA). Error bars indicate SD of triplicate determinations for subject S4. **B** When IL-1β was co-incubated with a 100-fold excess of IL-1ra [43], complete abrogation of the IL-1β effect on IL-8 and IL-6 upregulation was observed. The overall comparison using two-factor ANOVA analysis revealed significant differences (*p* < 0.001). Scheffé Test comparisons showed significant differences between Control vs IL-1β (p < 0.001) and IL-1β vs IL-1ra (*p* < 0.001). Error bars indicate SD of triplicate determinations. **C** Time-course of CCL5 ELISA response to IL-1β ± SP (24–72 h). The overall comparison using one-factor ANOVA revealed significant differences between groups (p < 0.001). Error bars indicate SD of triplicate determinations. **D** Time-course of TNF-α ELISA response to IL-1β ± SP (24–72 h). The overall comparison using one-factor ANOVA revealed significant differences between groups (*p* < 0.001). Error bars indicate SD of triplicate determinations. **E** Western blot showing that preincubation of P3 ESC with SP, for 1 h before addition of IL-1β (PSP), had the same inhibitory effect as simultaneous co-incubation of IL-1β plus SP (SSP) on IL-1β, IL-6, TNF-α and MMP3 reduction. SP alone reduced basal (control) SASP markers. β-Actin levels were not affected. **F** Western blot reveals inhibition of IL-1β stimulation of IL-1β, IL**-**6, MMP3, p21 and HMGB1 by JNK1 (CJ1) or JNK2 (CJ2) siRNA constructs transfected into P3 ESC. β-Actin levels were not affected.

ELISA of conditioned media confirmed findings in the Western blots. CCL5 secretion was upregulated by IL-1β in ESC derived from four subjects (S1, S2, S3, S4) and these effects were blocked by SP co-incubation (Fig. 4A). ANOVA showed significant upregulation of CCL5 from baseline after IL-1β-exposure in ESC from all four study subjects (*p* < 0.001), ANOVA with post-hoc Scheffé’s tests revealing significant inter-group differences (*p* < 0.001) between control *vs*. IL-1β-exposed ESC and IL-1β-exposed *vs*. IL-1β plus SP-treated cells). In experiments where IL-1β was co-incubated with a 100-fold excess of IL-1ra, complete abrogation of the IL-1β-induced upregulation of IL-8 and IL-6 was observed (Fig. 4B), suggesting that the effects were mediated via IL-1 receptor type 1 signaling. Two-factor ANOVA analysis revealed a significant difference in IL-6 and IL-8 (*p* < 0.001 for each). IL-1β time-dependently increased CCL5 and TNF-α, and SP suppressed CCL5 and TNF-α secretion after 24–72 h of IL-1β exposure (Fig. 4C, D, respectively). One-factor ANOVA analysis indicated that IL-1β stimulation induced IL-6 and IL-8 secretion, and this effect was completely blocked upon co-incubation with SP (*p* < 0.001 in each). The timing of addition of SP did not appear consequential. One h preincubation with the kinase inhibitor (PSP), had the same inhibitory effect as simultaneous co-incubation of IL-1β plus SP (SSP) on IL-1β, IL-6, TNF-α and MMP3 reduction (Fig. 4E). β-Actin levels were not affected. AS also suppressed IL-1β-induced CCL5 and TNF-α secretion, but its potency in ESC was only 43% that of SP (data not shown).

An alternative strategy to identify the responsible IL-1β signaling pathway was afforded using JNK1 and JNK2 siRNA interference. These constructs reduced IL-1β-induced IL-1β, IL-6, MMP3 and HMGB1 in ESC as determined by Western blotting (Fig. 4F) but had no effects on β-actin. Basal and IL-1β-stimulated p21 were inhibited with JNK2 siRNA but not as effectively by JNK1 siRNA (Fig. 4F). Pilot experiments using scrambled control siRNA showed no effects on IL-1β signaling (data not shown).

**Fig. 5:**
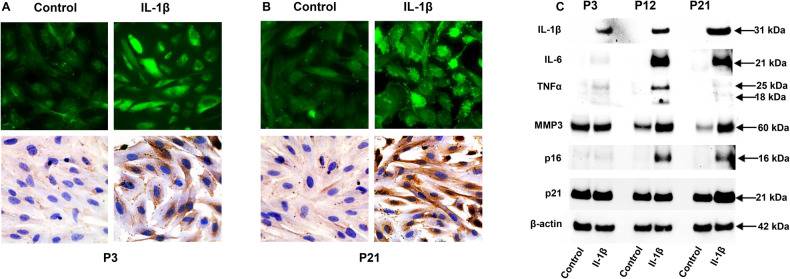
Effects of passage number on IL-1β stimulation. **A** Immunofluorescence- (IFC, top panels) and immunoperoxidase cytochemistry (IPC, lower panels) were used to localize IL-1β in ESC before and after IL-1β stimulation in P3 cells. **B** IFC (top panels) and IPC (lower panels) identified more IL-1β after IL-1β stimulation in P21 cells than P3 cells. Cell orientation in P21 cells also appears more parallel. **C** Western blot shows IL-1β, IL-6, TNF-α, MMP3, p16, p21 all increased with extended cell passage (P3 to P21) after incubation with IL-1β. β-Actin levels were unchanged.

**Fig. 6:**
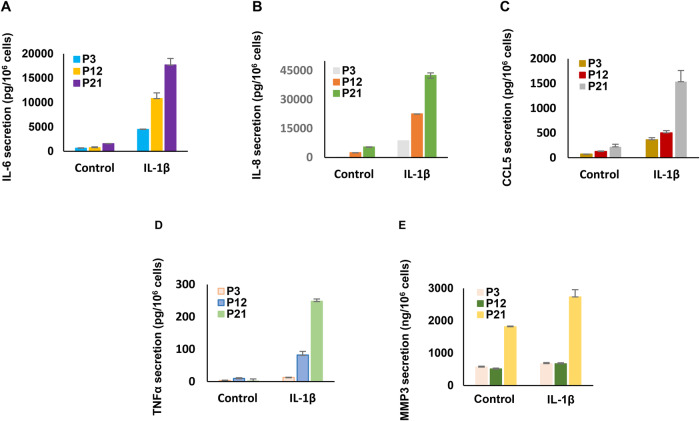
The upregulation of senescence-associated biomarkers was increased following extended cell passages, determined by ELISA assay. ELISA demonstrates that IL-6, IL-8, CCL5, TNF-α and MMP3 (**A**–**E**, respectively) were all increased with extended cell passage (P3, P12, and P21), and showed substantially enhanced responsiveness to IL-1β. Following IL-1β stimulation, a two-factor ANOVA analysis revealed a significant upregulation of five SASP components: IL-8 (*p* < 0.01), IL-6 (*p* < 0.001), CCL5 (*p* < 0.05), MMP3 (*p* < 0.01), and TNF-α (*p* < 0.001). For a more detailed description of the Scheffé Test analysis results, please refer to the Results section. Subtle but similar trends were noted under control conditions with increased biomarker concentrations in prolonged passage ESC. Error bars indicate SD of triplicate determinations.

The effects of cell passage number on IL-1β as an inflammaging marker were assessed in Fig. 5A, B by IFC and IPC. P21 ESC showed high basal and IL-1β-induced IL-1β accumulation (Fig. 5B) compared to P3 cultures (Fig. 5A). Western blots of ESC lysates from P3, P12 and P21 cells after IL-1β stimulation showed increased protein expression in higher passage number cells, except for β-actin (Fig. 5C). ELISA confirmed that IL-6, IL-8, CCL5, TNF-α and MMP3 were all progressively more responsive to IL-1β in P12 and P21 than in P3 cells (Fig. 6A–E). Two-factor ANOVA analysis indicated a significant upregulation of five SASP components following after IL-1β stimulation: IL-8 (*p* < 0.01), IL-6 (*p* < 0.001), CCL5 (*p* < 0.05), MMP3 (*p* < 0.01) and TNF-α (*p* < 0.001). Scheffé Test was utilized to compare between passages (P3 *vs*. P21) following IL-1β treatment: IL-6 (*p* < 0.01), IL-8 (*p* < 0.001), CCL5 (*p* < 0.05), TNFα (*p* < 0.01), and MMP3 (*p* < 0.001); Additionally, under control conditions, subtle yet similar trends were observed with prolonged ESC passage.

### Role of inflammation as an inhibitor of ESC differentiation

**Fig. 7:**
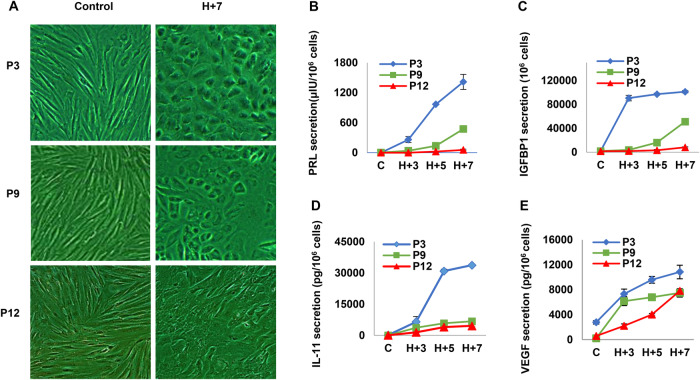
Decidual differentiation impaired in extended ESC passages. **A** Phase contrast microscopy shows fibroblastic cell morphology under control conditions (without added hormones, left panels) that is transformed to MET (cell “rounding”) after 7 days of hormone treatment (10 nM estradiol, 100 nM progesterone, 0.5 mM dibutyryl cAMP, H + 7, right panels). Low passage cells (≤ P9) have the most prominent hormone-induced cell shape changes, but these are inversely proportional to passage number, with MET effects more attenuated in hormone-treated P12 cells. Quantification of cell shape indices is provided in the text. Magnification×100. Biochemical markers of ESC differentiation quantified by ELISA included classical (**B** prolactin, **C** IGFBP-1; upper panels) and emerging (**D** IL-11, **E** VEGF; lower panels) decidual biomarkers. Concentrations in conditioned media from P3 (blue), P9 (red) and P12 (green) ESC, following three (H + 3), five (H + 5) or seven (H + 7) days incubation with or without (C, control) hormones are shown. Two-factor ANOVA unveiled a significant interaction in the overall comparison, signifying the upregulation of PRL, IGFBP1, IL-11, and VEGF (p < 0.001 for each). For a more detailed description of the Scheffé Test analysis results, please refer to the Results section. Error bars indicate SD of triplicate determinations.

Inflammation, particularly that mediated by IL-1β, can impair decidual differentiation in human ESC [23]. We postulated that prolonged passage number also may be associated with reduced ESC decidualization. ESC differentiation based upon cell shape was assessed by phase-contrast microscopy. Under control culture conditions (without added hormones) fibroblastic ESC morphology was indistinguishable among P3, P9 or P12 cells (Fig. 7A, left panels). Low passage cells ( ≤ P9) treated for seven days with a standardized decidualizing regimen (10 nM estradiol, 100 nM progesterone and 0.5 mM dibutyryl cAMP, H + 7) demonstrated cell “rounding” typical of MET [15]. The extent of MET in H + 7-treated ESC, reported as cell shape indices from 0.0 to 1.0, was inversely related to passage number. No significant differences in cell shape index were noted under control conditions (P3 = 0.18 ± 0.02, P9 = 0.40 ± 0.31, P12 = 0.29 ± 0.17, *P* = 0.19, 1-factor ANOVA). But following H + 7 hormone stimulation, statistically significant differences were observed among different ESC passage numbers (P3 = 0.95 ± 0.35, P9 = 0.64 ± 0.27, P12 = 0.43 ± 0.11, *p* < 0.01, 1-factor ANOVA). Post-hoc Scheffe’s tests revealed significant inter-group differences (*p* < 0.05) between hormone treated P3 *vs*. P9 cells and P3 vs. P12 cells. Two-factor ANOVA showed a significant interaction between the independent variables (passage number and days of hormone treatment) with F-values ranging from 12.6 to 99.9 (*p* < 0.001). Although attenuated at P12, H + 7 hormones still induced an increase in cell shape index over P3 controls without hormones (0.43 ± 0.11 vs. 0.18 ± 0.02, *p* < 0.01, Student’s *t-*test).

Cytological features of decidualization were associated with classical (prolactin, IGFBP-1) and emerging (IL-11, VEGF) biochemical correlates of ESC differentiation. ELISA quantified secreted biomarkers as continuous, dependent variables in conditioned media of P3, P9 and P12 ESC, under control (C) conditions and following three (H + 3), five (H + 5) or seven (H + 7) days of hormone exposure (Fig. 7B–E). Two-factor ANOVA showed a significant interaction between the independent variables (passage number and days of hormone treatment) with F-values ranging from 20.5 to 281.4 (*p* < 0.001) in all four decidualization biomarkers (Fig. 7B–E). The Scheffé test was utilized to compare control (C) versus seven days of hormone treatment (H + 7), C vs. H + 7, and within-group comparisons in PRL (Fig. 7B) revealed significant differences at P3 ( < 0.001), P9 (*p* < 0.001), and with no significant differences at P12 (*P* > 0.05). Further analyses using the Scheffe’s Test (H + 7) demonstrated significant inter-group differences at day seven post-hormone treatment (H + 7): P3 vs. P9 (*P* < 0.001), P3 vs. P12 (*P* < 0.001), and P9 vs. P12 (*P* < 0.01). Similarly, comparisons were conducted for IGFBP1 (Fig. 7C) using the Scheffé Test (H + 7), indicating significant inter-group differences in each comparison: P3 vs. P9, P3 vs. P12, and P9 vs. P12 (*P* < 0.001 for each). For IL-11 (Fig. 7D), significant differences were observed between P3 vs. P9 and P3 vs. P12 (*P* < 0.001), with no significant differences in P9 vs. P12 (*P* > 0.05). Finally, for VEGF (Fig. 7E), no significant differences were found in any of the comparisons: P3 vs. P9, P3 vs. P12, and P9 vs. P12 (*P* > 0.05 for each).

The results suggest notable variability in the response to hormone treatment across various passages of ESC, underscoring the heightened responsiveness of earlier passages (P3 and P9) compared to later passages (P12). Among the secreted biomarker proteins, all were most vigorously upregulated by hormones in P3 cells and their responsiveness was positively correlated with days of hormone exposure. The relative kinetics and magnitude of each biomarker were unique, with IGFBP-1 showing the largest differential response with respect to passage number (Fig. 7B) and VEGF having the smallest dynamic range (Fig. 7E). The results support the overall concept that primary ESC cultures lose hormone responsivity and differentiated function with extended passaging.

## Discussion

The effects of inflammation on markers of ESC aging were assessed using an established IL-1β treatment protocol [17] that mimics aspects of ESC senescence and endometriosis. In RNAseq experiments, IL-1β upregulated a number of bona fide SASP transcripts, indicated in Fig. 1A, B. Corresponding proteins paralleled their mRNAs, as shown by ELISA (MMP3, CCL5 and TNF-α, Fig. 1C, D) and IFC (p21, Fig. 2). Western blots (Fig. 3B, C) in cells treated with JNK inhibitors SP, AS or JNK1 and JNK2 siRNA implicated that pathway as key to IL-1β signaling (Figs. 3B, C and 4A–E). The EC_50_ of IL-1β actions in these cells [17, 27], is consistent with its affinity for the IL-1 receptor 1/IL-1 receptor accessory protein complex [28]. SASP gene products identified by RNAseq include many inflammatory biomarkers that have been historically analyzed in endometriosis, including IL-1β, IL-6 [29], IL-8 [30], CCL5 [31], TNF-α [32], CCL2 [33], MMP3 [34], HMGB1 [35], p16, and p21 [36, 37]. The findings support the senescence hypothesis proposed by Malvezzi et al. [37] for this condition.

Recent studies addressing senescence-induced pregnancy loss in mice reveal the involvement of similar cytokines and inflammatory biomarkers. Conditional knock-out of the uterine Enhancer of zeste homolog 2 (Ezh2) gene, a histone methyltransferase that epigenetically regulates decidual gene transcription, resulted in smaller embryo implantation sites and midgestational pregnancy failure. Decidual IL-1β and MMP3 transcripts were noted to be upregulated [38] and p16 and p21 proteins were elevated in this model [39].

The current experiments also demonstrate that hormone-induced ESC differentiation is compromised in a cell passage-dependent manner, manifested by temporal attenuation of MET morphology (Fig. 7A) and reduced secreted decidual biomarkers (prolactin, IGFBP-1, IL-11, and VEGF), *pari passu* as ESC passage number increased (Fig. 7B–E). The reliable utility of primary human ESC cultures as a model of decidual differentiation appears to be limited to the first six cell passages. A similar finding was published by Nayyar et al. [40], showing progressive reduction in IGFBP-1 secretion from menstrual effluent-derived ESC over weeks in culture. Also consistent with our observations, protein and mRNA biomarkers in ESC derived from clinically non-receptive patients in a Japanese IVF practice, exhibited significantly more senescence than ESC derived from subjects experiencing successful IVF pregnancies [41]. Based on these results we postulate that poor reproductive success (including failed implantation, miscarriage, and disorders associated with pathological placentation) are clinical manifestations of inflammaging.

Over the past decade, there has been significant advancement in understanding the influence of inflammation on infertility related to implantation failure. Traditionally, IL-1β, a key inflammatory mediator, was thought to be primarily produced by macrophages infiltrating the endometrium. Recent findings, however, suggest an alternative possibility, which is that IL-1β is notably auto-upregulated in ESC and not due to macrophage infiltration. We found that IL-1β levels in endometrial stromal cells (ESC) are low, often below the limits of detection by conventional methods like Western blotting. Remarkably, the introduction of human recombinant IL-1β markedly boosted the production of endogenous IL-1β by ESC themself, resulting in its exponential amplification in cultured ESC. In culture, exposure to IL-1β not only enhanced the release of IL-1β within ESC but also increased its reception by additional ESC. This led to a self-reinforcing feedback loop of IL-1β auto-upregulation, perpetuating a chronic inflammatory state. This pivotal discovery calls for a re-evaluation of the origins of IL-1β-associated inflammation and highlights the crucial role of ESC as participants in endometrial inflammation and receptivity. Further, our results suggest that inflammation may predispose to implantational delay or failure. The concept of “inflammaging,” which merges inflammation and aging, highlights this critical intersection, underscoring its significance for understanding the pathobiology of implantational failure and conditions that arise from poor placentation.

Brighton et al discovered striking similarities between cellular senescence and differentiation of ESC into decidual cells [42]. In our findings, we observed small increases in both IL-6 and CCL5 levels, each less than tenfold, after seven days of hormone treatment compared to controls (data not shown). In contrast, there was a greater than hundreds fold increase in IL-6 and CCL5 levels following 24 h of IL-1β treatment (Fig. 1C) [43, 44]. This significant divergence from hormone treatment highlights that acute decidual senescence plays a vital and necessary role in implantation, facilitating physiological repair and remodeling. In contrast, senescence induced by IL-1β may initiate persistent pathological changes that impair endometrial plasticity, reduce endometrial receptivity, and lead to poor implantation outcomes.

In this study, we made a novel observation, which is that IL-1β treatment led to a progressive rise in endogenous IL-1β expression levels in ESC throughout extended cell passages. To the best of our knowledge, this is the first description of this pattern. This intriguing pattern raises the question of whether there’s a correlation with heightened sensitivity to IL-1β stimulation, possibly linked to age-related alterations in cell generations, warranting further investigation.

Models, such as the one described here, and more complex 3-dimensional spheroids and organoids incorporating multiple cell types [45, 46], should inform further understanding of endometrial aging. We are hopeful that senescent changes associated with advanced maternal age might ultimately be reversed with properly adjudicated medicinal interventions, based on the IL-1β and JNK signaling pathways identified in this report.

## Materials/subjects and methods

### Sources of human tissues

Four participants undergoing elective laparoscopic surgery provided written informed consent. The study received ethics approval and consent to participate from the institutional review boards at Wake Forest School of Medicine, Winston-Salem, North Carolina (IRB #00019887). Patients were excluded if they did not read English, were unable to provide written informed consent, or were incarcerated, ensuring that informed consent was obtained from all subjects who fully understood the study. Fertile, parous subjects with regular menstrual cycles, ranging in age from 27 to 37 years, who had not received hormonal therapy for at least three months before surgery were recruited. Endometrial biopsies were collected in the proliferative phase by Pipelle aspiration under sterile conditions immediately prior to laparoscopy. Among the participants, no visible evidence of endometriosis, active infection or pelvic mass suspicious for malignancy was noted. The primary operative findings in one were intramural and subserosal fibroids, one had filmy periovarian adhesions, and two desired tubal sterilization without evidence of pelvic pathology.

### Endometrial stromal cell (ESC) preparation, culture conditions and in vitro decidualization

Endometrial biopsies were digested with collagenase and glands and debris were separated by filtration [47]. ESC, in the flow-through fraction, were sub-cultured twice to eliminate macrophages and other leukocytes by plating directly onto 10-cm polystyrene dishes at a density of 25,000 cells per cm^2^ in DMEM/Ham’s F-12 medium, supplemented with 10% fetal calf serum and antibiotics (penicillin and streptomycin). P3 ESC were studied unless noted otherwise. Further passages at 1:2 splits were performed as noted below, up to passage P21. In some experiments, cells were plated on Nunc Lab-Tek chamber slides (Thermo-Fisher Scientific, Waltham, MA) for immunofluorescence (IFC)- and immunoperoxidase-cytochemistry (IPC). ESC prepared in this manner are >93% pure and retain functional estrogen and progesterone receptors, as well as other phenotypic endometrial markers, for up to six passages (P6) in vitro, but tend to dedifferentiate with more protracted passaging [48].

Decidualization was induced in vitro by exposing the cultures to 10 nM 17β-estradiol + 100 nM progesterone + 0.5 mM dibutyryl cAMP in 0.1% EtOH (“hormones, H”) in phenol red-free medium (DMEM/Ham’s F-12 cat# 10-092 cv, CellGro, Manassas, VA, USA) supplemented with 5% charcoal-stripped (hormone-depleted) fetal calf serum to mimic the endocrine milieu of early pregnancy, as described [49]. Passage numbers of the cell preparations are indicated as P3, P9, P12 or P21. Control conditions were achieved by incubation in the same phenol red-free medium for seven days, but solvent alone (0.1% EtOH) was substituted for hormones. Morphological changes consistent with mesenchymal-epithelial transition (MET) were determined after phase contrast microscopy using cell shape index values ranging from 0.0 to 1.0 in 10 randomly selected ESC per photomicrographs as described [15, 50].

### IL-1β-treatment to recapitulate cellular inflammation and induce senescence

To mimic inflammatory aging, termed “inflammaging,” [26], ESCs were incubated in the presence of 0.1 nM human recombinant IL-1β (Sigma-Aldrich, St. Louis MO, cat# I9401), which we showed was the EC_50_ for several biomarker endpoints in ESC [17]. Cellular lysates for mRNA sequencing (RNAseq) and Western blotting and culture supernatants for ELISA were prepared after 24-72 h IL-1β exposure, as described below, and compared to untreated ESC cultures. IL-1ra (R&D, cat # 280-RA-010), a natural inhibitor that binds avidly to IL-1 type 1 receptors [51] was added at 10 nM (a 100-fold molar excess) to block IL-1 receptor signaling [17]. Pathway analyses were assessed using selective pharmacological c-Jun-N terminal kinase [JNK] inhibitors. Effects of exposure of ESC to IL-1β or IL-1β + SP600125 (“SP,” NSC 75890, Sigma, cat #S5567 [43]) or AS602801 (“AS,” bentamapimod, Sigma, cat# SML-3358), were compared after 24 to 72 h. A concentration of 30 μM was used for SP and AS, based on prior optimization in ESC [17]. As an alternative approach to block JNK action in ESC, JNK1 and JNK2 double-stranded siRNA constructs or a scrambled duplex control purchased from Cell Signaling Technologies (cat# 6232, 6233 and 6568), were introduced into ESC via transient transfection at a concentration of 100 nmol per well using Lipofectamine RNAiMAX (Thermo-Fisher) as described [17]. Successful inhibition of JNK1 and JNK2 protein expression was verified by Western blotting.

### RNAseq, transcriptome and statistical analyses

Total RNA was isolated from cell lysates using the TRIzol® Plus RNA Purification Kit (Life Technologies, Grand Island, NY). RNA concentrations and quality were measured by the Nanodrop 2000® method. cDNA was synthesized from mRNA samples and subsequently used as template for bulk RNAseq as described [52]. Per-cycle basecall (BCL) files generated by the Illumina NextSeq 500 were converted to per-read FASTQ files using bcl2fastq version 2.20.0.422 with standard default parameters. Sequencing quality was reviewed using FastQC version 0.11.9, detection of potential contamination was done using FastQ Screen version 0.14.1. FastQC and FastQ Screen quality reports were summarized using MultiQC version 1.9.0. RNA sequence accession codes are GSE261130 in NCBI tracking system.

No appreciable adapter sequences were detected so no trimming was performed. Genomic alignments were performed using HISAT2 version 2.2.1 with default parameters. NCBI reference GRCh38 was used for the reference genome and gene annotation set. Sequence alignments were compressed and sorted into binary alignment map (BAM) files using Samtools version 1.15.1. Counting of mapped reads for genomic features was performed using Subread featureCounts version 1.6.2 with the parameters “-s 2 –g gene_name –t exon –Q 60 -B -C”. The annotation file specified with—a was the NCBI GRCh38 reference provided by Illumina iGenomes. Alignment statistics and feature assignment statistics were again summarized using MultiQC.

Differentially expressed genes were detected using the Bioconductor package DESeq2 version 1.34.0. Genes with one count or less were filtered, and alpha was set to 0.05. DESeq2 tests used a negative binomial generalized linear model, dispersion estimates, and logarithmic fold changes. DESeq2 calculates log2 fold changes and Wald test *P* values, performs independent filtering and adjusts for multiple testing using the Benjamini-Hochberg procedure to limit false discovery rate (FDR) to *p* < 0.05. Volcano plots confirmed the upregulation of SASP gene transcripts, and they were corroborated at the protein level by immunocytochemistry, ELISA and Western blots.

### Immunofluorescence- (IFC) and Immunoperoxidase-cytochemistry (IPC)

To verify the cellular localization of biomarker proteins, ESC were plated on Lab-Tek® chamber sides and grown to 80% confluence. Some cultures were stimulated with 0.1 nM recombinant IL-1β for 24 h, followed by extensive washing. ESC were fixed in 4% paraformaldehyde and permeabilized with ice cold acetone. The cells were stained with β-actin to label cytoskeleton microfilaments (cat# 3700, Cell Signaling, 1:600 antibody dilution). SASP and DNA damage biomarkers included IL-1β, p21. The suppliers, conditions and concentrations of antibodies used for IFC and IPC are described in Antibody Table 1. In IPC experiments nuclei were counterstained with hematoxylin.

**Table 1:** Antibody table.

Target of Antibody/siRNA	Manufacturer, Catalog No.	Dilution Used	Tested applications	Species Raised in	RRID
IL-1β	Cell Signaling Technology,12703	1:1000 and 1:400	WB, IFC	R-M	AB_2798278
IL-1β	Cell Signaling Technology,12242	1:1000 and 1:100	WB, IPC	M-M	AB_2715503
IL-6	Cell Signaling Technology,12153	1:1000	WB	R-M	AB_2687897
TNFα	Cell Signaling Technology, 6945	1:1000	WB	R-M	AB_10859375
MMP3	Cell Signaling Technology,14351	1:1000	WB	R-M	AB_2798459
p16	Cell Signaling Technology, 92803	1:1000	WB	R-M	AB_2750891
p21	Cell Signaling Twchnology,2947	1:1000 and 1:400	WB, IFC	R-M	AB_823586
HMGB1	Cell Signaling Technology, 6893	1:1000	WB	R-M	AB_10827882
CCL2	Cell Signaling Technology, 39091	1:1000	WB	R	AB_2799147
CCL5	Cell Signaling Technology, 2987	1:1000	WB	R	AB_2244129
Phospho-Histone H2A.X	Cell Signaling Technology, 9718	1:1000	WB	R-M	AB_2118009
β-Actin	Cell Signaling Technology,3700	1:1000 and 1:400	WB, IFC	M-M	AB_2242334
SignalSilence SAPK/JNK siRNAI	Cell Signaling Technology, 6232	100 nM	Transfection		
SignalSilence SAPK/JNK siRNAII	Cell Signaling Technology, 6233	100 nM	Transfection		

#### Senescence-associated-β-galactosidase (SAβG) staining

SAβG staining was conducted on confluent endometrial stromal cells (ESC) in 6-well plates using the Senescence β-Galactosidase Staining Kit (#9860; Cell Signaling Technology, MA, USA), in strict accordance with the manufacturer’s instructions. ESC were seeded onto 6-well plates and cultured until they reached 50–60% confluence. Selected cultures were then stimulated with 0.1 nM recombinant IL-1β for 24 h. Following this, cells were fixed in 1× fixative solution for 10–15 min at room temperature and stained overnight with 2 ml of β-galactosidase staining solution at 37 °C in a dry incubator (no CO_2_). To prevent evaporation, plates were sealed. The next day, the staining solutions were removed, and the cells were overlaid with 70% (v/v) glycerol in water and stored at 4 °C. The staining results were visualized using light microscopy, and images were captured.

### ELISA for detection of SASP biomarkers and in vitro decidualization

Sensitive and specific sandwich ELISA kits for senescence biomarkers, IL-6, IL-8, TNFα, MMP3 and CCL5 (cat# D6050, D8000C, TDA00D, DMP300 and DRN00B, R&D Biosystems, Minneapolis, MN), were measured to define the effects of IL-1β on ESC cultures. ELISA also were used to quantify secreted biomarkers of ESC decidualization: prolactin (cat# 0300, Alpha Diagnostic International, San Antonio, TX); IGFBP-1, VEGF, and IL-11 (cat# DGB100, DVE00, and D1100, R&D Biosystems, Minneapolis, MN). We and others previously established these assays to be precise and linear in ESC and they are increasingly recognized as reliable biochemical surrogates for ESC decidualization [11, 12, 49].

### Western blot analysis

To quantify intracellular protein expression, Western blots were performed on whole-cell lysates collected in extraction buffer (cat# FNN0011, Life Technologies, Grand Island, NY), followed by protein quantification using the Thermo Scientific-Pierce BCA™ Protein Assay method (cat# PI-23227, Thermo-Fisher Scientific, Waltham, MA) [15]. 30 μg protein per lane were loaded onto NuPAGE® Novex® 4-12% Bis-Tris protein gels (Thermo-Fisher), electrophoresed, and transferred to PVDF membranes. After blocking with 5% skim milk in PBS, primary antibodies to senescence biomarkers (IL-1β, IL-6, IL-8, TNF-α, MMP3, p16, p21, HMGB1, CCL2, CCL5 and phospho-γ-histone 2 A.X (listed in Antibody Table 1) were incubated overnight and the blots were then exposed to secondary goat anti-rabbit or anti-mouse antibodies (1:300 ,000; cat# 31460 and 31430, Pierce Biotechnology Inc., Rockford, IL) linked to horseradish peroxidase and visualized by SuperSignal™ West Pico PLUS (cat# PI34577, Thermo Scientific). Blots were washed, re-probed with mouse monoclonal anti-human β-actin antibodies (1:1000 dilution, cat# 3700, Cell Signaling) to confirm evenness of loading. SeeBlue Plus2® pre-stained molecular weight standards (cat# LC5925, Life Technologies, Grand Island, NY), were used to calibrate migration of proteins. The molecular mass of each band is indicated at the right of each Figure as kDa. Full-length copies of the original Western gels are provided as a single Supplemental Fig. 1.

### Data presentation and statistical analyses

Results and statistical evaluation of RNAseq and transcriptome findings were assessed as described above. Data from biomarker ELISA and cell shape indices were all found to be normally distributed (Kolmogorov-Smirnov tests) and are presented as means ± SD when performed in triplicate. Each experiment was repeated a minimum of three independent times with representative findings shown. SASP biomarkers in our model of ESC inflammation were compared by 1-factor ANOVA in control ESC *vs*. those stimulated with IL-1β in the absence or presence of SP (NSC 75890), AS (bentamapimod) or JNK1 or JNK2 siRNA. Post hoc Scheffé’s tests were used to compare inter-group differences among the SP-, AS- or siRNA-treated samples. Comparisons among cell shape indices and ELISA data for decidual markers were made using 2-factor ANOVA, with passage number and hormone treatment as the categorical independent variables. Cell shape indices compared among different passage numbers with or without hormone treatment were analyzed by 2- and 1-factor ANOVA. 10 randomly selected ESC photomicrograph images were used for cell shape measurements under each experimental condition. Statistical significance was accepted when two-tailed tests yielded *p* < 0.05.

## Availability of data and materials

All datasets will be made available to readers promptly upon request.

### Supplementary information

Fig3.A

Fig3.B

Fig3.C

Fig4.E

Fig4.F

Fig5.C

Merged original no cropped EB dat
