# Supplementary figures and images for: Interleukin-1β induces and accelerates human endometrial stromal cell senescence and impairs decidualization via the c-Jun N-terminal kinase pathway
Source: Cell Death Discov. 2024 Jun 15;10:288. doi: 10.1038/s41420-024-02048-6 (PMC11180092; doi:10.1038/s41420-024-02048-6)

IL-1 $\beta$

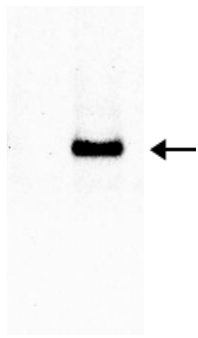

IL-6

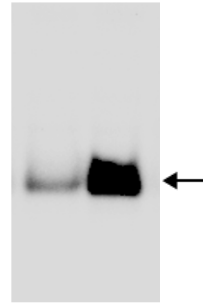

TNF $\alpha$

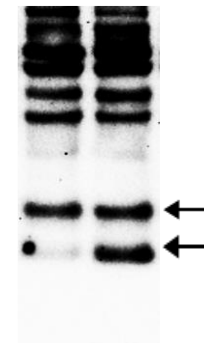

MMP3

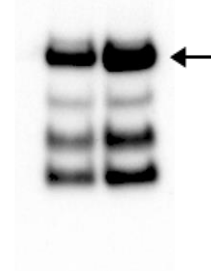

p16

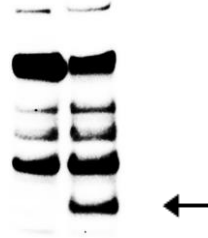

p21

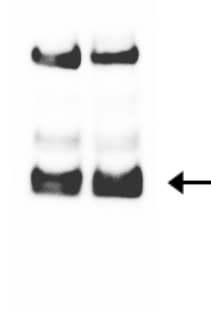

CCL2

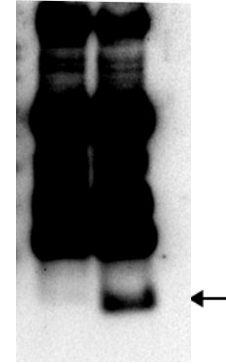

P-Histone-H2A.X

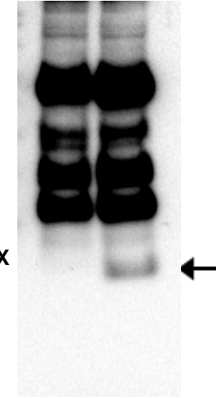

$\beta$ -actin

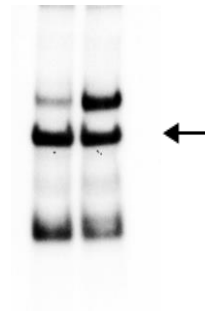

Supplement: Supplementary file 1 — Fig3.A [file 41420_2024_2048_MOESM1_ESM.pdf]

IL-1 $\beta$

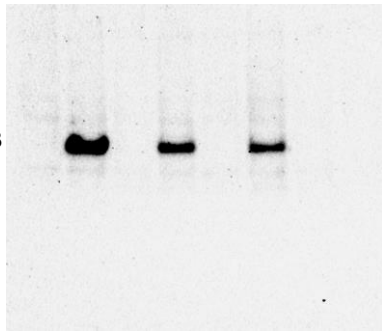

IL-6

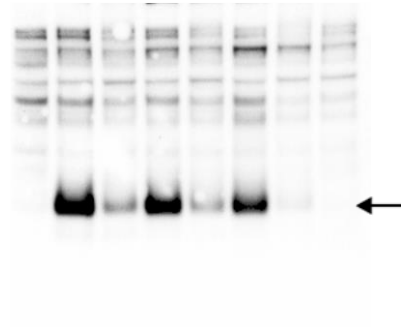

TNF $\alpha$

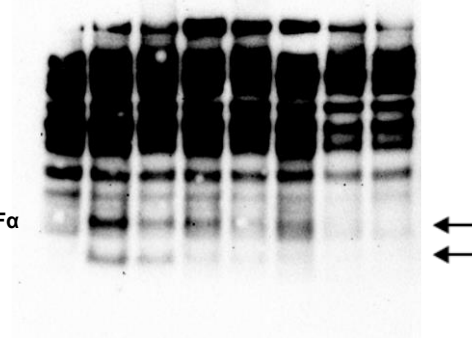

MMP3

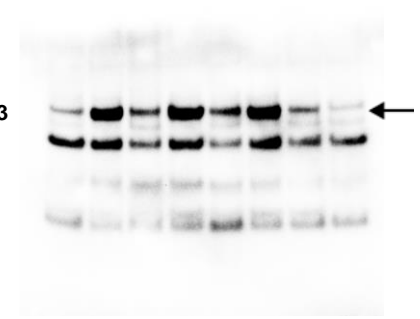

P16

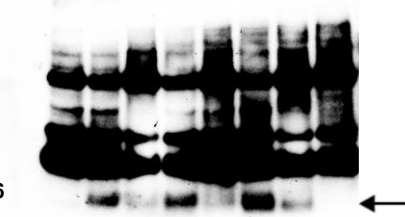

P21

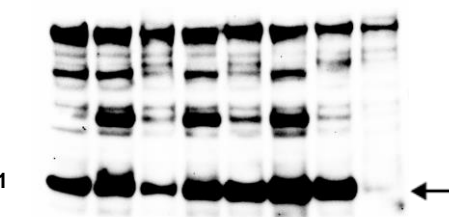

HMGB1

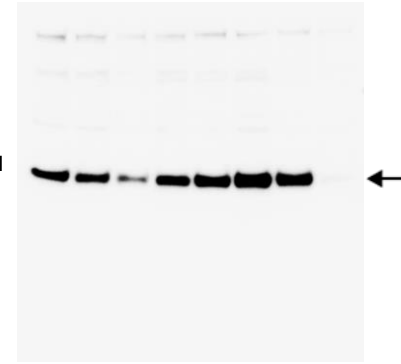

CCL2

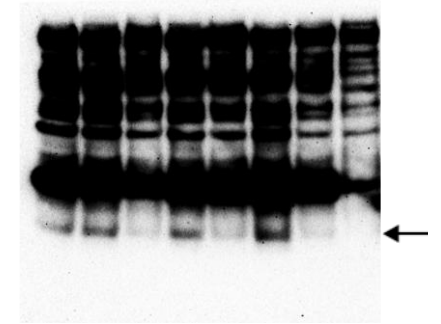

P-Histone H2A.X

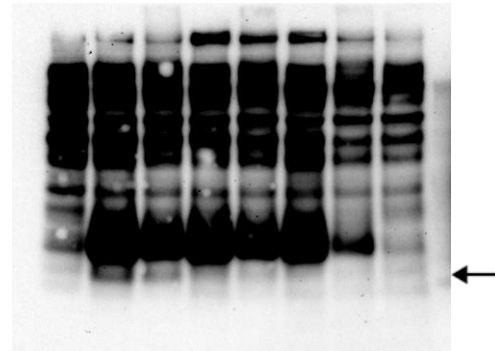

$\beta$ -actin

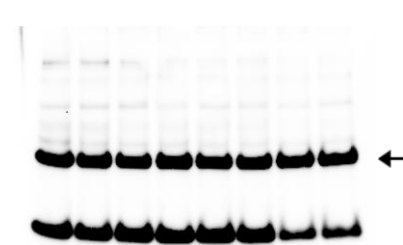

Supplement: Supplementary file 2 — Fig3.B [file 41420_2024_2048_MOESM2_ESM.pdf]

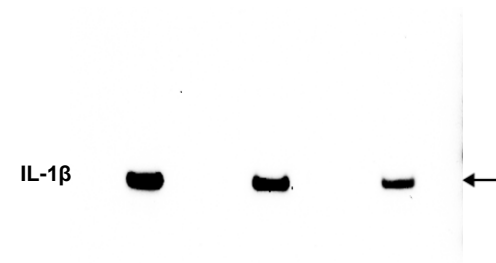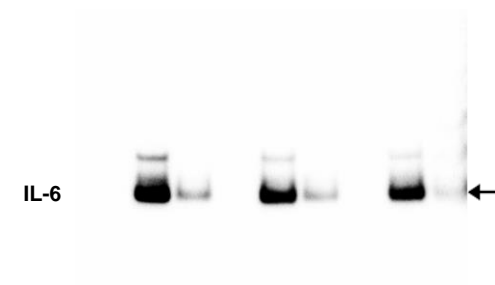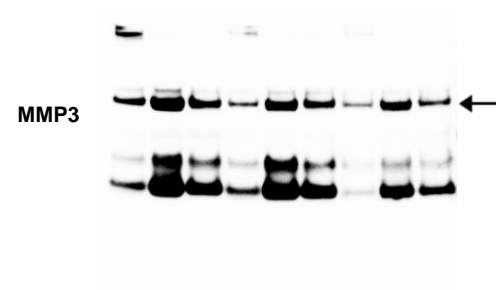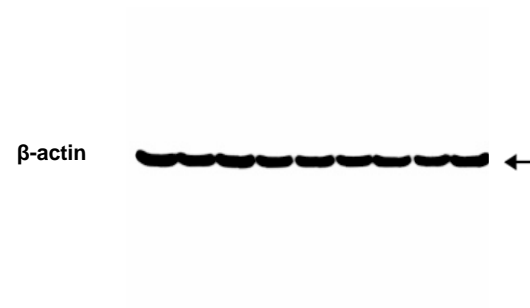

Supplement: Supplementary file 3 — Fig3.C [file 41420_2024_2048_MOESM3_ESM.pdf]

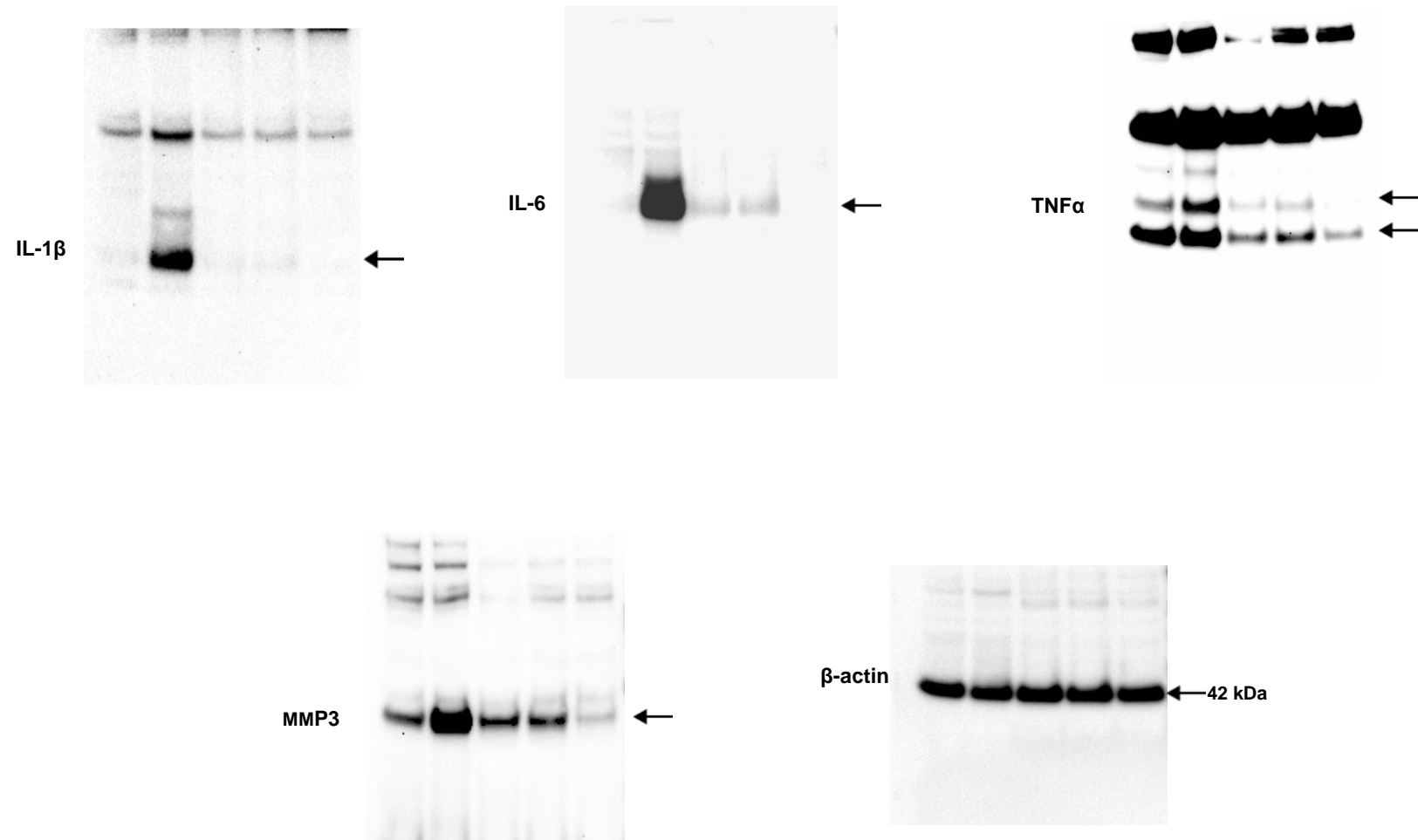

Supplement: Supplementary file 4 — Fig4.E [file 41420_2024_2048_MOESM4_ESM.pdf]

IL-1 $\beta$

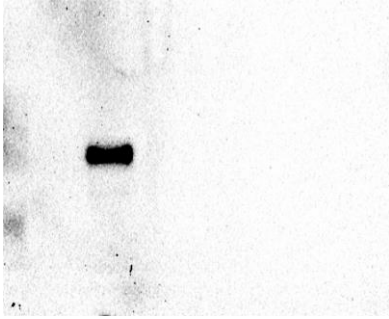

IL-6

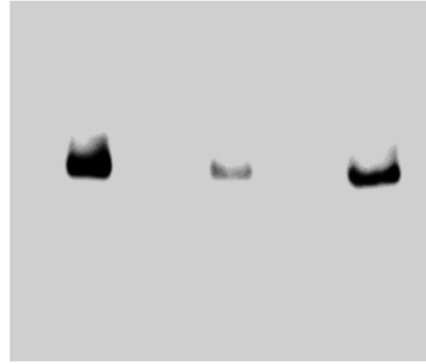

MMP3

p21

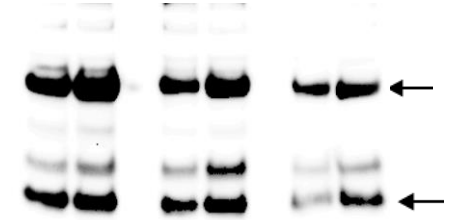

HMGB1

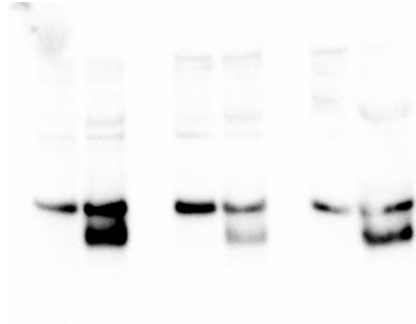

$\beta$ -actin

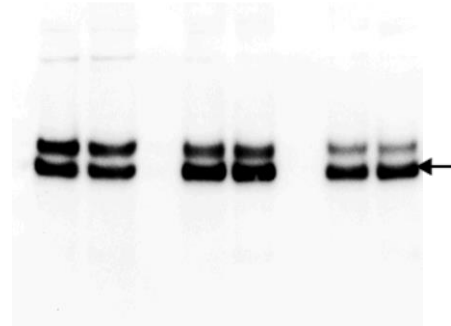

Supplement: Supplementary file 5 — Fig4.F [file 41420_2024_2048_MOESM5_ESM.pdf]

IL-1 $\beta$

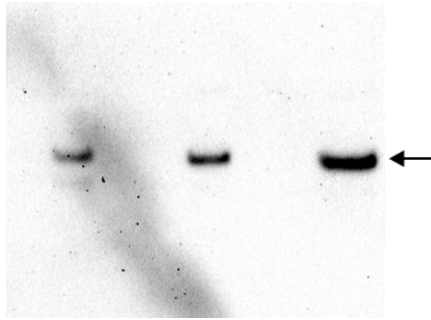

IL-6

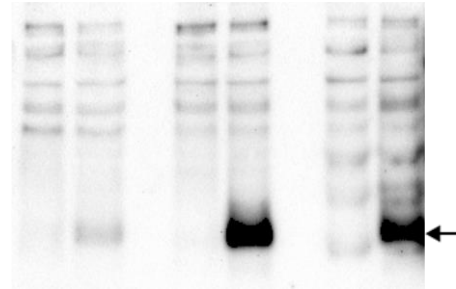

TNF $\alpha$

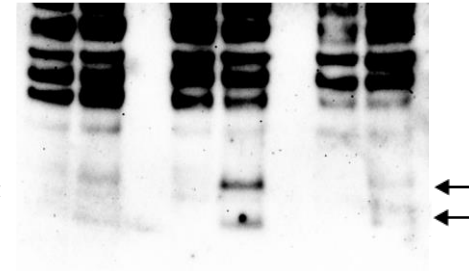

MMP3

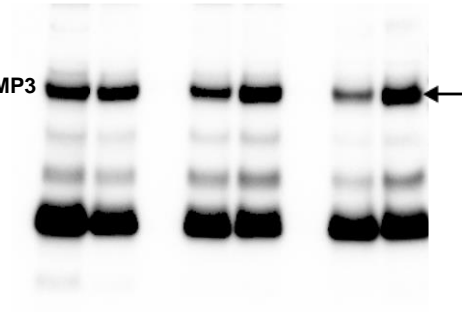

p16

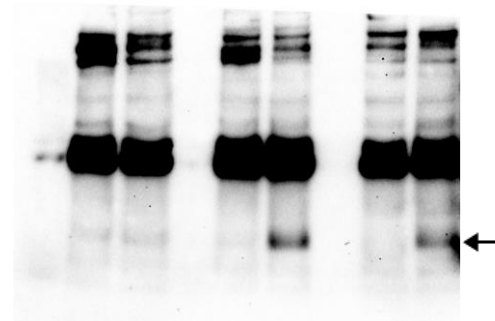

p21

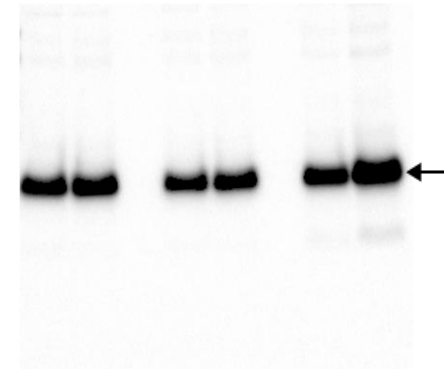

$\beta$ -actin

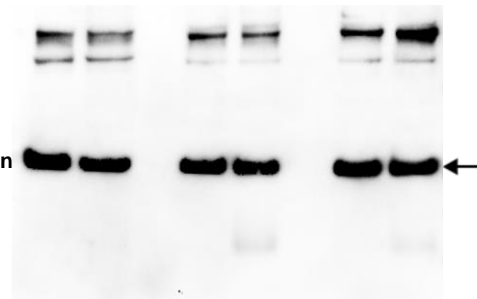

Supplement: Supplementary file 6 — Fig5.C [file 41420_2024_2048_MOESM6_ESM.pdf]

Fig.3 A

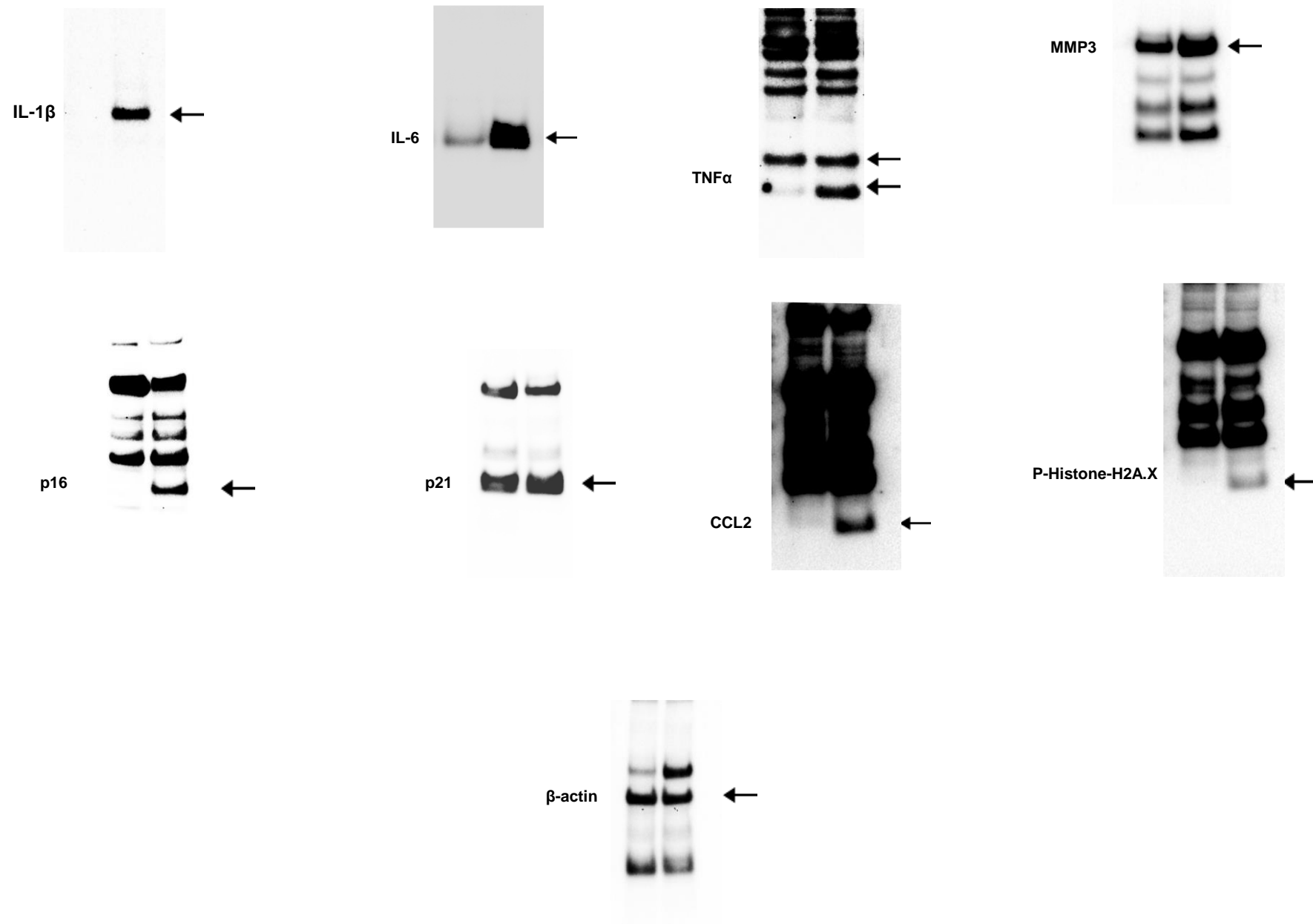

Fig.3B

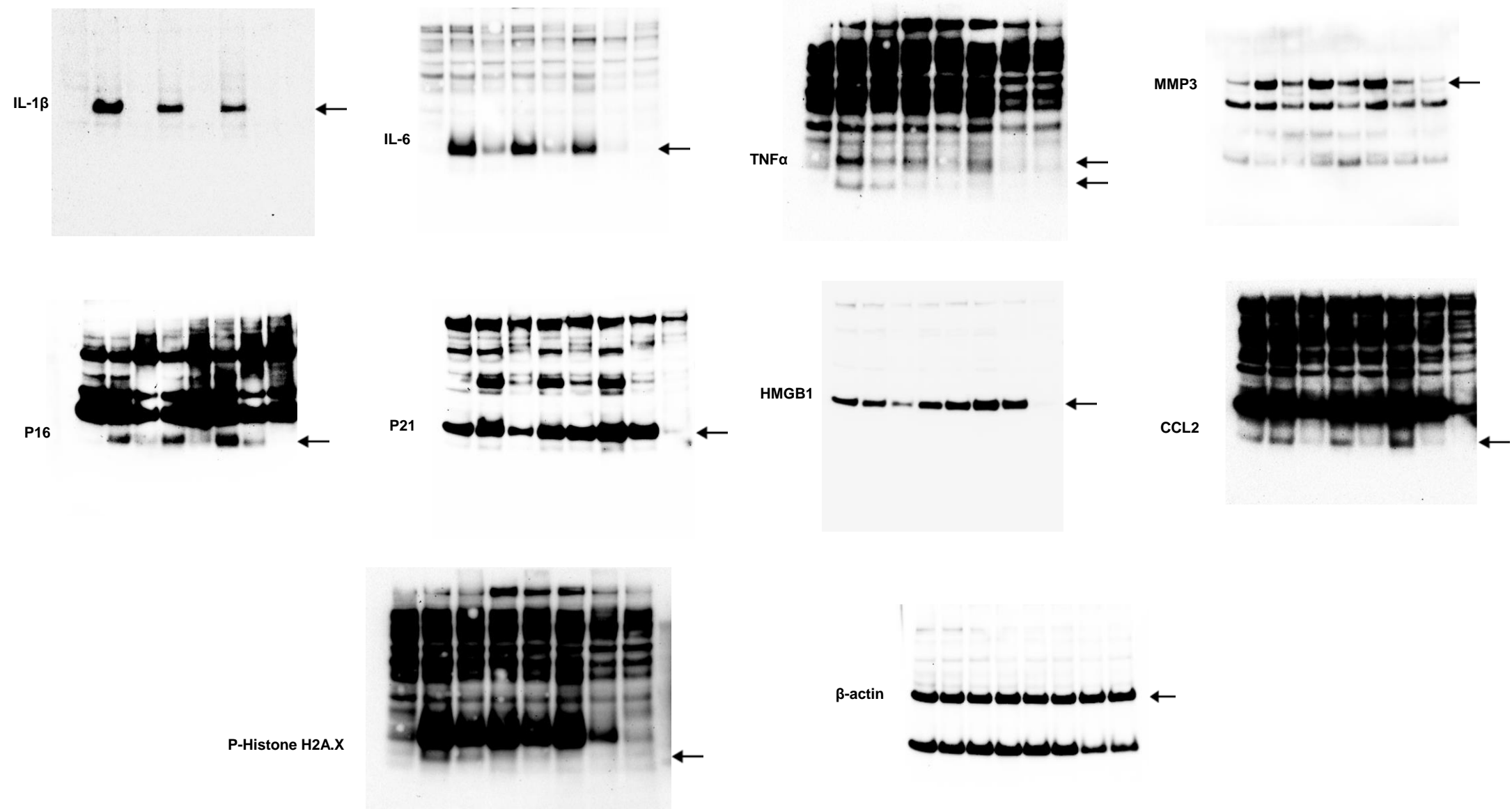

Fig.3C

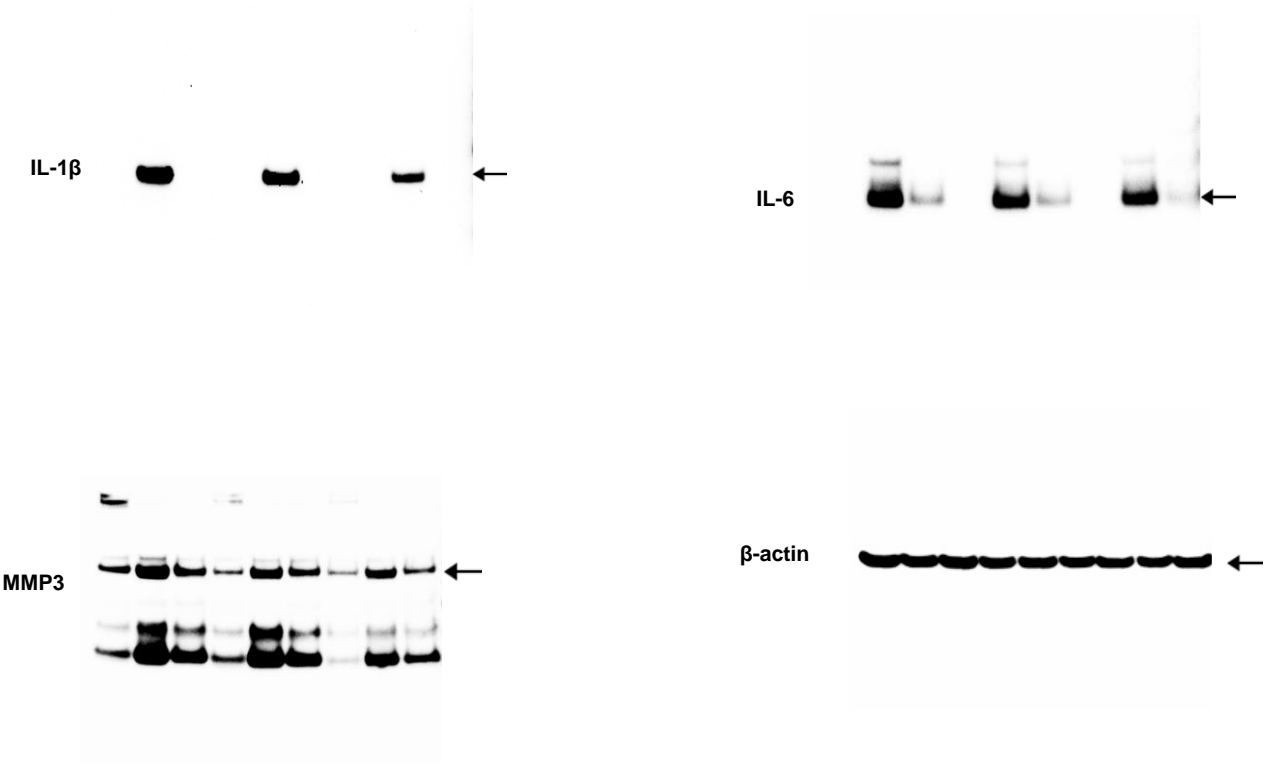

Fig.4E

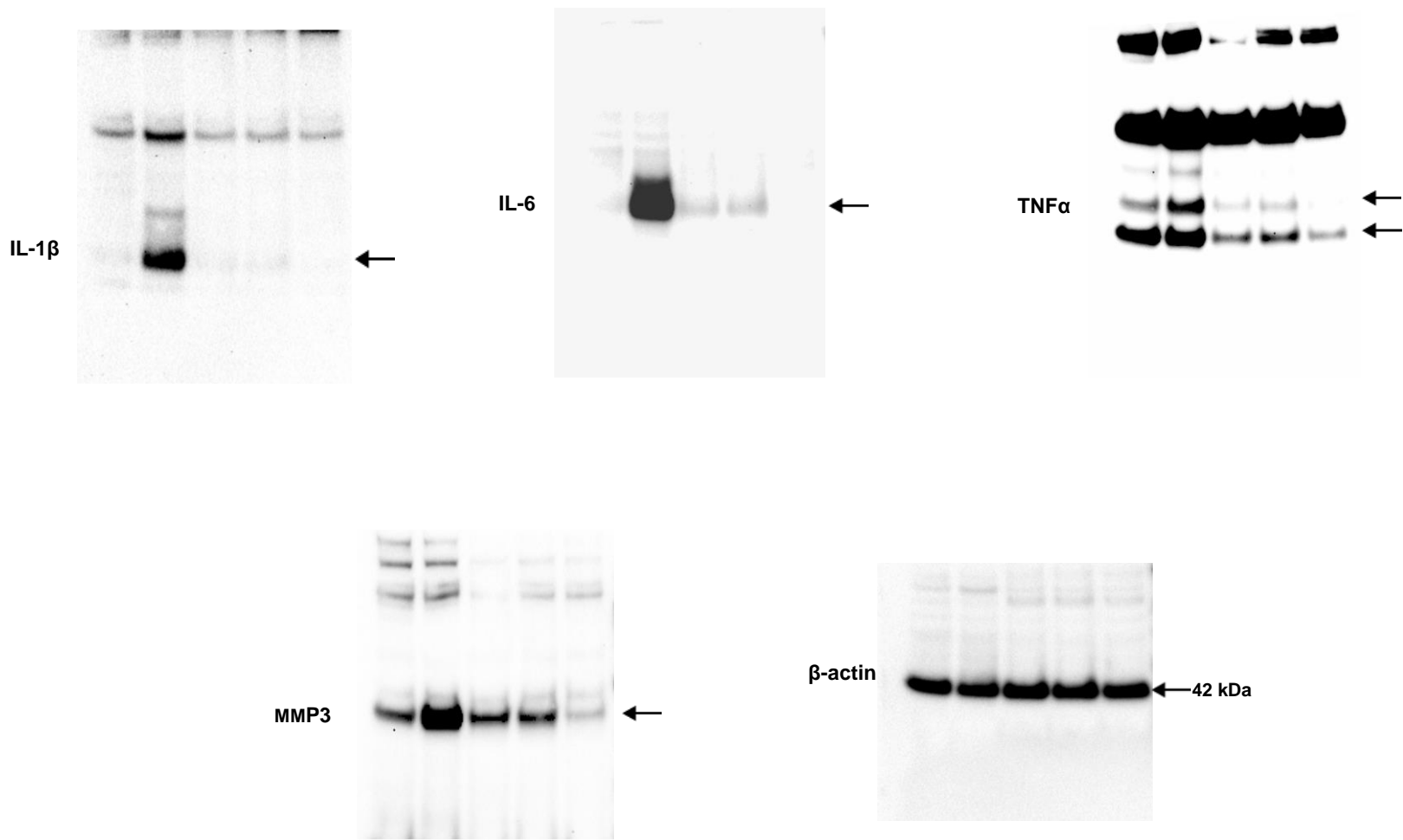

Fig.4F

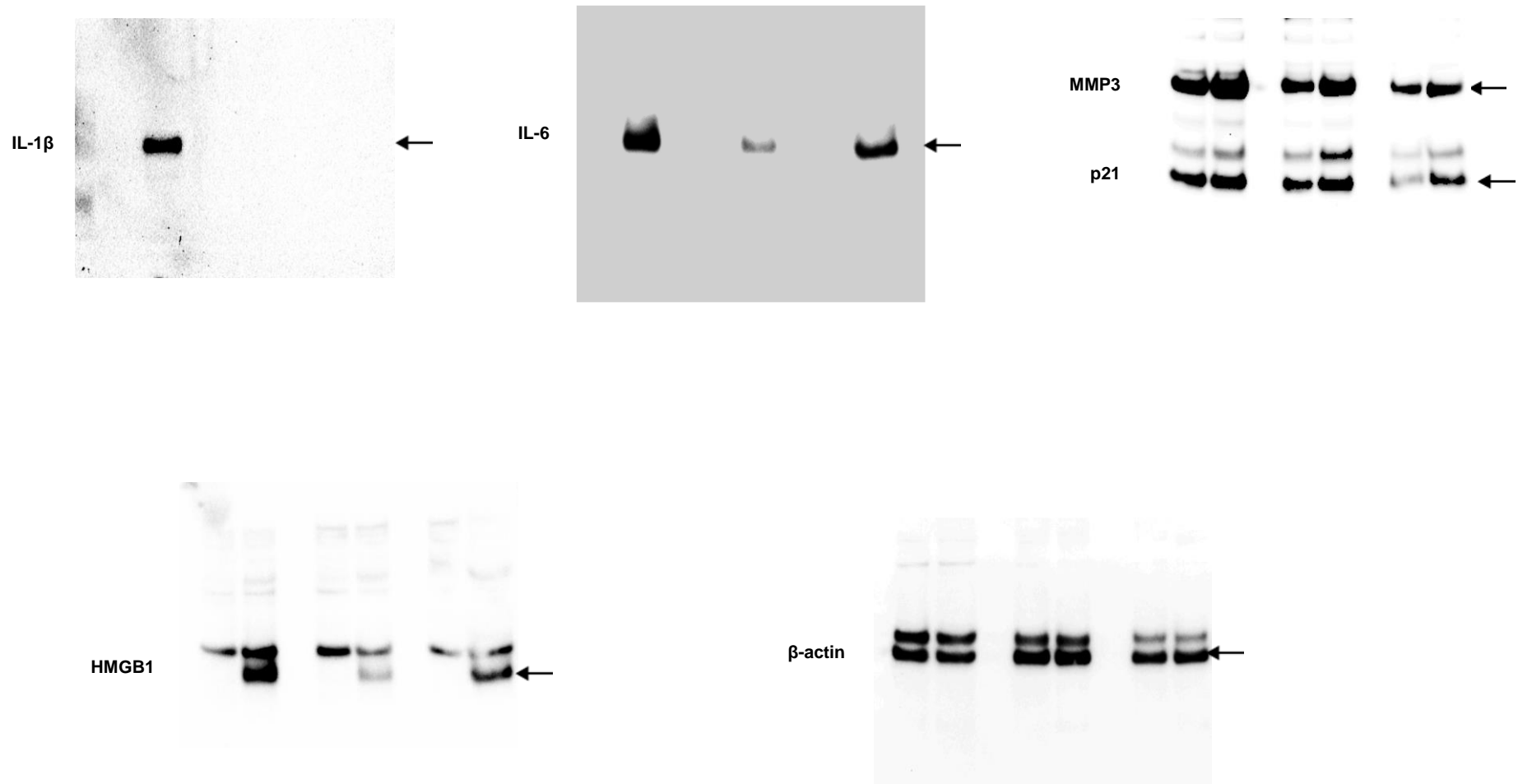

Fig.5 C

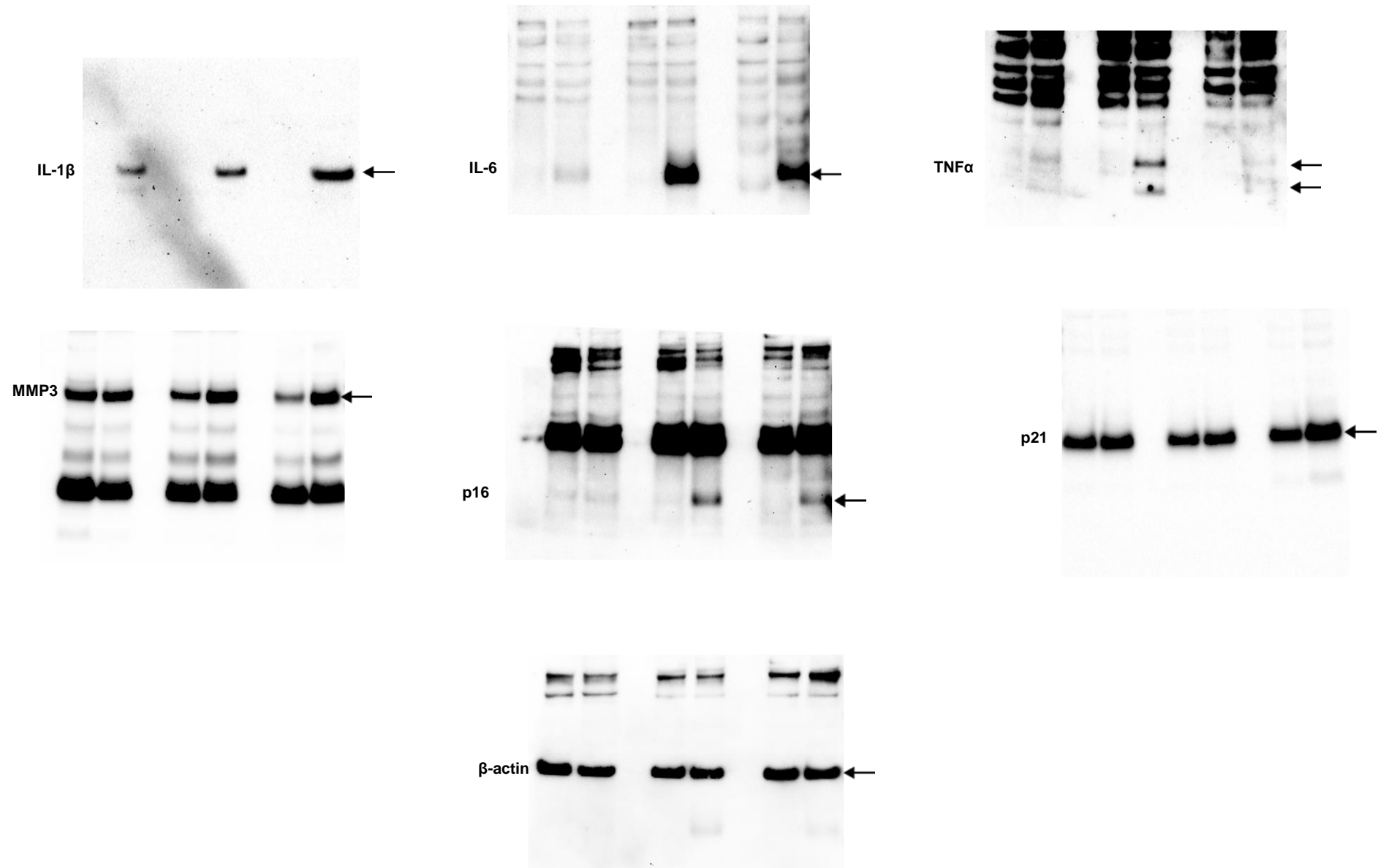

Supplement: Supplementary file 7 — Merged original no cropped EB dat [file 41420_2024_2048_MOESM7_ESM.pdf]
